# Attitudes and Commitment Toward Precautionary Measures Against COVID-19 Amongst the Jordanian Population: A Large-Scale Cross-Sectional Survey

**DOI:** 10.3389/fpubh.2021.745149

**Published:** 2021-11-08

**Authors:** Moawiah Khatatbeh, Hindya O. Al-Maqableh, Samir Albalas, Sara Al Ajlouni, Ashraf A'aqoulah, Haitham Khatatbeh, Mohammed A. Kasasbeh, Ibdaa Khatatbeh, Rahaf Albalas, Ala'a B. Al-Tammemi

**Affiliations:** ^1^Department of Basic Medical Sciences, Faculty of Medicine, Yarmouk University, Irbid, Jordan; ^2^Department of Health Service Administration, Yarmouk University, Irbid, Jordan; ^3^Department of Health Systems Management, College of Public Health and Health Informatics, King Saud Bin Abdulaziz University for Health Sciences, Riyadh, Saudi Arabia; ^4^King Abdullah International Medical Research Center, Riyadh, Saudi Arabia; ^5^Doctoral School of Health Sciences, University of Pecs, Pecs, Hungary; ^6^School of Nursing, Faculty of Health Sciences, Higher Colleges of Technology, Fujaira, United Arab Emirates; ^7^Department of Family and Occupational Medicine, Faculty of Medicine, University of Debrecen, Debrecen, Hungary; ^8^Doctoral School of Health Sciences, University of Debrecen, Debrecen, Hungary

**Keywords:** COVID-19, pandemic, Jordan, face mask, hand washing, PPEs, NPI

## Abstract

**Aims:** This study aimed to (1) assess the non-pharmaceutical intervention (NPI) measures that were used by the Jordanian population against COVID-19, and (2) determine the sociodemographic and behavioral predictors of contracting COVID-19 with a focus on the utilization of personal precautionary measures.

**Methods:** A descriptive questionnaire-based cross-sectional survey was used in this study. A structured web-based questionnaire was disseminated to the Jordanian community through social media platforms. Participants were asked a series of questions about socio-demographic characteristics, in addition to the knowledge, attitudes, and commitment toward using various personal precautionary measures (e.g., face mask, hand washing, social distancing) against the COVID-19. Data were analyzed using descriptive statistics, cross-tabulation, and binary logistic regression through SPSS^®^.

**Results:** Responses from 7,746 participants were included in our final analyses. Descriptive statistics showed that most participants (82.6%) believed that face mask protects against COVID-19. Around 69.5% of the participants were completely committed to wearing a face mask, while 65% of the participants were completely committed to hand washing. The results of the regression analysis revealed that female gender (AOR = 1.2; 95% CI: 1.07–1.35; *p* = 0.002), having a family member infected with COVID-19 (AOR = 8.5; 95% Cl: 7.51–9.70; *p* = 0.001), having a health-related work or study (AOR = 1.2; 95% Cl: 1.09–1.38; *p* = 0.001), believing that face masks do not protect against COVID-19 (AOR = 1.3; 95% Cl: 1.12–1.47; *p* = 0.001), and partial commitment to handwashing (AOR = 1.2; 95% Cl: 1.11–1.75; *p* = 0.006) were all associated with an increased odds of contracting COVID-19 among the participants.

**Conclusion:** Overall, commitment to non-pharmaceutical intervention (NPI) measures, such as wearing a face mask, hand washing, and physical distancing, was not optimal among Jordanians. This might explain the dramatic increase in the infectivity rate of the COVID-19 virus in the past few months in the country. More sustainable efforts regarding health promotion and strict policies are required to prevent a third wave of hitting the country and to prevent similar infectious threats in the future.

## Introduction

On 11 March 2020, the World Health Organization (WHO) declared that the coronavirus disease 2019 (COVID-19) outbreak had become a pandemic (1). As of 07 September 2021, severe acute respiratory syndrome coronavirus 2 (SARS-CoV-2) has infected more than 221 million individuals worldwide and caused over 4.5 million deaths. The primary route of transmission of COVID-19 is via respiratory particles (2). Since the 14th century, protective face-covering equipment has always been recommended during respiratory pandemics (3). Despite early vaccine development and the advances in treatment protocols, using facemasks and other non-pharmaceutical intervention measures (NPI) remain a priority to reduce disease transmission (4, 5). Furthermore, the US Centers for Disease Control and Prevention (CDC) recommends maintaining hand hygiene inside and outside households. Hand washing and sanitization are recommended as one of the most effective ways for personal and family protection against COVID-19 infection (6). Also, it is well-known that social distancing is one of the main factors affecting the spread of infectious diseases (7). Literature shows that household transmission had an immense contribution to the initial reproductive number after social distancing than before social distancing measures (8), while it is recommended to maintain 1.8 meters of distance from an infected household member, which might not be easily applicable (6).

In Jordan, family is considered to be the fundamental social unit (9). People in Jordan are religiously and socially committed to family values; they place a lot of importance upon social relationships and interactions. In other words, most Jordanian people live in close family settings and are expected to be closely socializing on a daily basis. This poses a threat for household transmission of COVID-19 as crowded indoor environments with close contact are considered particularly high-risk (10).

At the beginning of the COVID-19 crisis, Jordan's response to the pandemic was a truly unique experience, because the early and timely strict measures taken by the government have put Jordan at the forefront of middle east countries fighting the COVID-19 pandemic in terms of the number of cases (1, 11, 12).

On 26 January 2020, the Jordanian National Epidemic Committee, and the Jordanian Ministry of Health (MoH) had met to put a plan in place to encounter the pandemic. The MoH had equipped designated hospitals with ventilators, personal protective equipment (PPE), and enforced all healthcare workers for using PPEs and quarantine policies (13). On the 2nd of March 2020, Jordan has implemented strict measures after the first confirmed case was reported. As of 17 March 2020, the government enacted the Defense Law, activating a state of emergency to contain an outbreak of the coronavirus pandemic. Defense Order number 26/2021 under the provisions of Defense Law umber 13/1992, the first section was issued as follows: “Every person must adhere to the established physical distances and adhere to wearing a mask before entering public places,” further solidifying the rule of the PPEs with an application of penalty-system (14). Also, media has been heavily utilized to alert Jordanians of the severity of COVID-19 infection and the speed of its spread, and Jordan has been considered a model country in facing the pandemic (1, 15, 16). Citizens were made more aware of the need for social distancing and the importance of using PPEs.

This social adjacency of Jordanians, along with the flattened epidemic curve, encouraged authorities to alleviate lockdown measures and encouraged people to adopt less strict preventive measures. As a result of this alleviation, Jordan changed from a role model in combating the pandemic to one of the most impacted countries with a severe physical and psychological burden (16–18). According to Jordan's MoH data, more than 803 351 cases were confirmed across the country as of 07 September 2021 (19).

Despite the rapid expansion in the literature and available information on media outlets regarding COVID-19, some aspects of the disease have not been identified yet. The ambiguity about the pandemic leads to an enormous amount of misinformation and rumor culture (20). There is a small yet growing group of people who refuse to wear PPEs or take vaccinations; this can be attributed to psychological reactance and conspiracist sentiment (21–23).

Previous studies have widely covered the use of NPI measures among healthcare subgroups such as physicians, nurses, and medical students in Jordan (23–27). To the best of our knowledge, literature about the Jordanian general population is still limited concerning the use of NPI measures. Also, the available data about the Jordanian population's handwashing habits amidst the pandemic are limited. Accordingly, this study aimed to (i) assess and explore the NPI measures used by the Jordanian population against COVID-19 and the commitment to using various PPE, and (ii) determine the sociodemographic and behavioral predictors of contracting COVID-19 with a focus on the utilization of personal precautionary measures (handwashing, using facemasks, and social distancing). The results of this study are expected to inform the public health policymakers in Jordan about the key predictors of COVID-19 transmission in the community. Such information will also help in controlling the subsequent pandemic waves with a better preparedness and response plan.

## Methods and Materials

### Study Setting

Our study was conducted in Jordan, an Arabic-speaking country located in the WHO Eastern Mediterranean Region (EMR) with a population count of nearly 10.8 million.

### Study Design and Participants

A descriptive questionnaire-based cross-sectional survey was employed in this study focusing on Jordan's general population. A convenience sample was recruited through social networking platforms through the snowball sampling technique. A web-based (online) questionnaire was firstly developed in English, then it was translated into Arabic (the native language of our respondents) by two bilingual specialists using translation and back-translation techniques. The web-based questionnaire was created using Google Form^®^ and was disseminated to those who could access the online survey (i.e., internet users in Jordan). The inclusion criteria included being a Jordanian citizen aged 18 or older, capable of reading and understanding Arabic, and willing to fill the online questionnaire. The questionnaire, along with an introductory letter about the study and its objectives and eligibility criteria, was sent to participants via social media platforms like Facebook^®^, WhatsApp^®^, LinkedIn^®^, in addition to email addresses, for a period of 2 months (March–April 2021). In addition, respondents were asked to share the questionnaire's link with their friends and family members via their social network, employing exponential non-discriminative snowball sampling technique. The authors did not provide any incentives or rewards upon participation; thus, submitting more than one response was unlikely.

### Study Instrument and Measures

The authors have reviewed available and relevant literature (References) and developed a 21-item questionnaire that comprised two main sections. The first section consisted of eight questions on participants' socio-demographic characteristics such as age (in years), gender (male, female), education (up to secondary education, undergraduate education, postgraduate education), the field of work or study (health-related, non-health-related), history of contracting COVID-19 infection (yes, no), having a family member diagnosed with COVID-19 (yes, no), number of persons living at home/residence place (4 or less, 5–7, more than 7), and number of persons sleeping in one room (1, 2, 3 or more).

The second section comprised 13 items that solicited data about knowledge, attitudes, and commitment toward using PPEs and other precautionary measures. In this section, the participants were requested to report the following: type of face-mask used (surgical, non-surgical), how often a face mask is changed in a day (never, once, twice, ≥3 times), what type of face masks the participant think or believe to be more protective against COVID-19 (surgical, non-surgical), level of commitment to wearing face mask (not committed, partial, complete), the participant's opinion regarding the public's commitment to wearing face masks (not committed, partial, complete), the participant's opinion regarding the proper way of wearing face masks (covering nose and mouth, covering mouth only, covering nose only). Additionally, the participants were asked to report their opinion whether a face mask protects against COVID-19 or not (yes, no), if wearing a face mask and gloves together increases protection against COVID-19 (yes, no), if wearing two face masks together increases protection against COVID-19 (yes, no), and if wearing a face mask and socially distancing together increase protection against COVID-19 (yes, no). Also, participants were asked to report their overall level of commitment to handwashing (not committed, partial, complete), whether the participant practiced handwashing after touching the elevator's button (yes, no), and whether the participant practiced handwashing after touching money (yes, no).

### Content and Face Validity

Two researchers checked the content of the questionnaire and its face validity before the final approval. To ensure its reliability, the questionnaire was pilot tested with the first 30 responses (18 females and 12 males, with mean age of 36 years). Based on these responses and the feedback, refinements were made accordingly. The Cronbach's alpha score was found to be 0.84. Also, the answers from pilot-testing were not included in our final analyses.

### Ethical Considerations

All participants have given their informed consent through reading the following statement and ticking a box next to it: “*Completing the questionnaire would be considered consent to voluntary participation*” The study was reviewed and approved by Yarmouk University's Institutional Review Board (IRB) Committee (IRB/2021/39). Participants were informed that the study would not disclose any personal information and that their data would be stored under high-security settings with only the research team having access to the data.

### Statistical Analysis

The Statistical Package for Social Sciences (SPSS- IBM, Chicago, IL, USA) was used to analyze the data. Categorical variables were reported as frequency counts and percentages. Also, a cross-tabulation analysis using the chi-square test was employed to assess significant differences between categorical variables. Significant factors revealed from the cross-tabulation analysis were subjected to a backward Wald stepwise binary logistic regression analysis (with the status of COVID-19 infection as a binary outcome variable) to assess the independent effect of each factor after controlling for potential confounders. A *p*-value < 0.05 was set for statistical significance.

## Results

A total of 7,746 complete responses were received from all governorates of Jordan (after excluding the pilot-phase responses). Two-thirds of the study population (67.6%) were females, and more than half of the participants (56.5%) were young adults aged 18–29 years. Interestingly, about one-third of the participants (32.9%) had a COVID-19 infection. Detailed socio-demographic characteristics of the study population are shown in Table 1.

**Table 1:** Socio-demographic characteristics of the study population (*n* = 7,746).

**Characteristic**	***n* (%)**
**Gender**	
Male	2510 (32.4)
Female	5236 (67.6)
**Age (years)**	
18–29	4,378 (56.5)
30–49	2,842 (36.7)
50–64	472 (6.1)
>65	54 (0.7)
**Your work or study field is health-related**	
No	5,747 (74.2)
Yes	1,999 (25.8)
**Education**	
Secondary or less	1,167 (15.1)
Undergraduate	5,254 (67.8)
Postgraduate	1,325 (17.1)
**Number of persons living in your house**	
≤ 4	3,035 (39.2)
5–7	3,720 (48.0)
>7	991 (12.8)
**Number of persons sleep in your room including you**	
1	1,867 (24.1)
2	4,383 (56.6)
≥3	1,496 (19.3)
**Diagnosis of COVID-19 in the family**	
No	3,279 (42.3)
Yes	4,467 (57.7)
**Diagnosis of COVID-19**	
No	5,198 (67.1)
Yes	2,548 (32.9)

Commitment and attitudes of the study population toward wearing a face mask and using other protective measures were assessed in this study. Despite the majority of participants (82.6%) believed that face mask protects against COVID-19 the rest of the participants (17.4%) didn't believe in that. Regarding the commitment to wearing face masks, only 69.5% of participants were completely committed to wearing a face mask. Moreover, about 65% of the study sample was completely committed to hand washing. Table 2 illustrates the results of the commitment and attitudes of the study population toward face masks and other protective measures.

**Table 2:** Commitment and attitudes of the study population toward various protective and precautionary behaviors (*n* = 7,746).

**Characteristic**	***n* (%)**
**Type of face mask you use**	
Surgical	4,889 (63.1)
Non-surgical	2,857 (36.9)
**How often do you change your face mask in a day?**	
Once	2,907 (37.5)
Twice	1,159 (15.0)
≥3	490 (6.3)
I do not change it	3,190 (41.2)
**Which type of face mask is more protective against COVID-19?**	
Surgical	5,083 (65.6)
Non-surgical	2,663 (34.4)
**What is your commitment level in wearing a face mask?**	
Completely committed	5,387 (69.5)
Partially committed	2,189 (28.3)
Not committed	170 (2.2)
**In your opinion, what is people's commitment level in wearing a**	
**face mask?**	
Completely committed	561 (7.2)
Partially committed	5,230 (67.5)
Not committed	1,955 (25.2)
**In your opinion, what is the correct way of using a face mask?**	
Covering nose and mouth	7,607 (98.2)
Covering mouth only	52 (0.7)
Covering nose only	87 (1.1)
**In your opinion, does a face mask protect against COVID-19?**	
No	1,350 (17.4)
Yes	6,396 (82.6)
**In your opinion, does wearing a face mask and gloves together**	
**increase protection against COVID-19?**	
No	2,294 (29.6)
Yes	5,452 (70.4)
**In your opinion, does wearing a face mask and distancing together**	
**increase protection against COVID-19?**	
No	326 (4.2)
Yes	7,420 (95.8)
**In your opinion, does wearing two face masks together increase**	
**protection against COVID-19?**	
No	3,161 (40.8)
Yes	4,585 (59.2)
**What is your commitment level to handwashing?**	
Completely committed	5,094 (65.8)
Partially committed	2,425 (31.3)
Not committed	227 (2.9)

To assess the association between having COVID-19 infection and other variables, a cross-tabulation analysis was performed. As expected, having a family member diagnosed with COVID-19 was strongly correlated with contracting COVID-19 infection (*p*-value < 0.001). Complete commitment to wearing a face mask and handwashing was less associated with having the infection (*p*-value < 0.05). The results of this analysis are shown in Table 3 and contain only the associations with a *p*-value < 0.25.

**Table 3:** Cross-tabulation of factors associated with contracting COVID-19 infection.

**Variable**	**COVID-19 infection**		***p*-value**
	**No (%)**	**Yes (%)**	
Gender			0.001
Male	1,751 (69.8)	759 (30.2)	
Female	3,447 (65.8)	1,789 (34.2)	
**Do you wash your hands after touching elevator buttons?**			0.186
No	1,443 (66.0)	744 (34.0)	
Yes	3,755 (67.5)	1,804 (32.5)	
**Do you wash your hands after touching money?**			0.168
No	2,120 (66.2)	1,081 (33.8)	
Yes	3,078 (67.7)	1,467 (32.3)	
**What is your commitment level to wearing a face mask?**			0.044
Completely committed	3,879 (72.0)	1,751 (28.0)	
Partially committed	1,450 (66.2)	739 (33.8)	
Not committed	112 (65.9)	58 (34.1)	
**What is your commitment level to handwashing?**			0.006
Completely committed	3,481 (68.3)	1,613 (31.7)	
Partially committed	1,567 (64.6)	858 (35.4)	
Not committed	150 (66.1)	77 (33.9)	
**Diagnosis of COVID-19 in the family**			<0.001
No	2,944 (89.8)	335 (10.2)	
Yes	2,254 (50.5)	2,213 (49.5)	
**Your work or study field is health-related**			0.001
No	3,916 (68.1)	1,831 (31.9)	
Yes	1,282 (64.1)	717 (35.9)	

The binary regression analysis revealed that female gender (AOR = 1.2; 95% CI: 1.07–1.35; *p* = 0.002), having a family member infected with COVID-19 (AOR = 8.5; 95% Cl: 7.51–9.70; *p* = 0.001), having a health-related work or study (AOR = 1.2; 95% Cl: 1.09–1.38; *p* = 0.001), believing that face masks do not protect against COVID-19 (AOR = 1.3; 95% Cl: 1.12–1.47; *p* = 0.001), and partial commitment to hand washing (AOR = 1.2; 95% Cl: 1.11–1.75; *p* = 0.006) were all associated with an increased odds of contracting COVID-19 among the participants. Table 4 presents the results of the binary logistic regression analysis.

**Table 4:** Logistic regression analysis of factors associated with an increased odd of contracting COVID-19.

**Variable**	**Adjusted odds** **ratio (AOR)**	**95% confidence interval**		***p*-value**
		**Lower**	**Upper**	
**Gender**				
Male	1^*^			
Female	1.2	1.07	1.35	0.002
**Does mask protect against COVID-19 infection?**				
Yes	1^*^			
No	1.3	1.12	1.47	0.001
**COVID-19 diagnosis among family**				
No	1^*^			
Yes	8.5	7.51	9.70	0.001
**Work or study is health-related**				
No	1^*^			
Yes	1.2	1.09	1.38	0.001
**How committed are you to handwashing?**				
Completely committed	1^*^			
Partially committed	1.2	1.11	1.75	0.006
Not committed	1.3	1.07	1.36	0.002

* *Reference for other categories*.

## Discussion

The current study explored the general public's attitudes and commitment toward various NPI measures against the COVID-19 in Jordan. Additionally, the study assessed several demographic and behavioral factors that were associated with a higher likelihood of contracting COVID-19. The regression model revealed that female participants had slightly and significantly higher odds of being infected with COVID-19 than male counterparts (OR = 1.2; *p* = 0.002). This finding was incongruent with the global literature which showed males as more susceptible to COVID-19 than females in terms of infectivity, severity, and mortality (28–30). The reasons behind males' vulnerability for worse outcomes might include immune-suppressive effects of testosterone, higher smoking rates, and higher rates of respiratory tract infections among males (31). Nevertheless, psychological distress and anxiety were reported to be higher among Jordanian females during the pandemic crisis (17, 32), and this may play a vital role in the vulnerability of females to contract COVID-19 infection, considering the proven link between psychological stressors and their negative impact on immune system's defense mechanisms against viral infections (33).

In the ongoing COVID-19 crisis, commitment to the imposed preventative and precautionary measures against infection is pivotal among healthcare workers and the general public (1, 24, 25). Face masks were recommended as one of the measures to mitigate the viral spread in the community. In our study, participants who believed that face masks do not offer protection against COVID-19 were found to have higher odds of catching COVID-19 A variety of face masks are available in the global market for use (34). Face masks were reported in the literature to have protective effects and reduce viral transmissibility between individuals (2, 35), especially when combined with other measures such as frequent hand washing/sanitization and physical distancing. A systematic review of 172 observational studies concluded that face masks can reduce the transmission of COVID-19 (35). In the United States, a recent survey aimed at assessing people's beliefs and attitudes toward mask-wearing and COVID-19 revealed that the majority of respondents believed that face masks help in reducing the transmission of the SARS-CoV-2 virus between people (36).

Additionally, individuals who had a COVID-19-infected family member quarantined in the residence place were found to have more likelihood of contracting COVID-19; that is, those individuals had statistically significant higher odds to be infected compared to the others with no family history of COVID-19 infection. Household transmission of COVID-19 was documented among pre-symptomatic, asymptomatic, and clinically symptomatic individuals (37), which reflects the importance of getting vaccinated against COVID-19 (38).

During the early stages of the pandemic, stringent control measures were enforced in Jordan, including total country lockdown with the round-the-clock nationwide curfew. These measures have contributed to the slow pace of viral transmission in the community due to limited physical contact (23, 33), However, the gradual opening of economic sectors that started in early June 2020, along with people's reduced compliance to COVID-19 preventative behaviors have led to a significant viral spread in the Jordanian community (15, 18).

Our result showed that the participants working or studying within the health field had a statistically significant higher chance of becoming infected than others. This result is consistent with a previous study which found that the front-line healthcare personnel had a higher risk of SARS-CoV-2 infection than the general public (39, 40).

Hand washing has numerous health benefits, including protection against influenza, coronavirus, and other infectious diseases. Our study showed that the participants who were not committed to handwashing are more likely to contract COVID-19 than those who were partially or completely committed. This indicates that commitment to hygiene measures is associated with a lower risk of COVID-19 infection amongst the general public. In a study that was conducted among the public in Saudi Arabia, hand washing was perceived by the respondents as the most effective measure to minimize the risk of COVID-19 infection (41). However, estimating the public's commitment to many COVID-19 preventive behaviors is challenging as it is subjected to social desirability while responding to these kinds of questions (42).

Our study findings shed the light on the importance of sufficient adoption of protective measures against the COVID-19. Yet, there are limitations that should be acknowledged while interpreting our results, and this includes, the cross-sectional nature of our survey in which there is generally no evidence of a temporal relationship between exposure and outcome, implementing an online questionnaire which could limit participation to only those who have sufficient digital literacy and access to the internet, utilizing a convenience sampling which limits the sample representativeness and generalizability of our findings, the questionnaire represents self-reported states; thus, recall bias and social desirability might influence the results, and lastly, the nature of using social media platforms to recruit research participants is challenging concerning participants who could possibly not fulfill eligibility criteria but still can submit a response, or those who may submit multiple responses. However, to overcome this issue, we gave explicit instructions to the participants that only those who meet the eligibility criteria can participate in the survey to maintain credible and reliable findings. Furthermore, there were no incentives or rewards upon participation. Consequently, participation of non-eligible individuals or submitting more than one response was unlikely.

Overall, commitment to precautionary measures, such as wearing a face mask, hand washing, and physical distancing, was not optimal among Jordanians. This can explain the dramatic increase in the infectivity rate of the COVID-19 virus in the past few months in the country. More sustainable efforts regarding health promotion and strict policies are required to prevent another wave of hitting the country and to prevent similar infectious threats in the future.

## Data Availability Statement

The raw data supporting the conclusions of this article will be made available by the authors, without undue reservation.

## Ethics Statement

The studies involving human participants were reviewed and approved by IRB, Yarmouk University, Irbid, Jordan. The participants provided their written informed consent to participate in this study.

## Author Contributions

MK, HA-M, AA-T, SA, and SAA: conceptualization. MK, HA-M, SA, SAA, and IK: methodology. MK, HK, IK, MAK, and AA-T: formal analysis. MK, HA-M, SA, SAA, AA, IK, RA, and AA-T: data curation. MK, HA-M, SA, SAA, AA-T, RA, AA, and MAK: writing—original draft preparation and writing-review and editing. MK: project administration. All authors contributed to the article and approved the submitted version.

## Funding

The publication of this article was supported by the Deanship of Scientific Research and Graduate Studies at Yarmouk University.

## Conflict of Interest

The authors declare that the research was conducted in the absence of any commercial or financial relationships that could be construed as a potential conflict of interest.

## Publisher's Note

All claims expressed in this article are solely those of the authors and do not necessarily represent those of their affiliated organizations, or those of the publisher, the editors and the reviewers. Any product that may be evaluated in this article, or claim that may be made by its manufacturer, is not guaranteed or endorsed by the publisher.
